# Sjögren syndrome induced by anti PDL-1 treatment for TNBC: case report and review of literature

**DOI:** 10.3389/fimmu.2024.1417444

**Published:** 2024-10-07

**Authors:** Caterina Pellegrino, Chiara D’Antonio, Debora Ierinò, Concetta Elisa Onesti, Anna Maria Aschelter, Daniele Santini, Federica Mazzuca

**Affiliations:** ^1^ Medical Oncology Unit, Sant’ Andrea Hospital of Rome, Rome, Italy; ^2^ Sarcomas and Rare Tumors Departmental Unit, IRCCS Regina Elena National Cancer Institute, Rome, Italy; ^3^ Department of Pathology, Oncology and Radiology, Sapienza University of Rome, Rome, Italy

**Keywords:** immunorelated adverse events, Sjogren syndrome, triple negative breast cancer, immune checkpoint inhibitors, case report

## Abstract

**Background:**

Rheumatological toxicity associated with immunotherapy, particularly Sjögren’s syndrome (SjS), has been observed with variable incidence in patients treated with immune checkpoint inhibitors (ICIs). Although SjS is a well-known autoimmune disease, its occurrence as an immune-related adverse event (irAE) during cancer treatment is less well understood. Current literature documents a range of incidence rates and clinical manifestations of SjS in patients undergoing ICI therapy, highlighting the need for early diagnosis and multidisciplinary management.

**Case presentation:**

A 40-year-old woman underwent mammography, which revealed a 43 mm mass in the left breast. Core biopsy confirmed grade 3 infiltrating triple negative ductal carcinoma with high MIB-1. She received neoadjuvant chemotherapy, followed by surgery and radiotherapy. A CT scan in September 2022 showed lung nodules and lymph node involvement. A lung biopsy confirmed breast cancer metastasis. She started treatment with atezolizumab and nab-paclitaxel with evidence of a partial response. Nab-paclitaxel was discontinued due to side effects and atezolizumab was continued as maintenance therapy. After four cycles, the patient developed symptoms consistent with Sjögren’s syndrome (SjS), which were confirmed by diagnostic tests. Treatment with prednisone, pilocarpine and hydroxychloroquine was initiated alongside ongoing immunotherapy. The patient continues to receive atezolizumab with stable disease and good quality of life.

**Conclusion:**

This case highlights the importance of recognizing SjS as a potential irAE in patients treated with ICIs, particularly those with TNBC. Multidisciplinary collaboration is essential for the prompt diagnosis and effective management of SjS to maintain both cancer control and patient quality of life. Given the recent emergence of these events and the lack of specific guidelines, our case report may provide valuable insights into the management of a little-known adverse event and pave the way for further real-world data collection on the management of these rare but significant toxicities that impact on patient quality of life. Further research is needed to optimize treatment protocols and outcomes for patients experiencing rheumatological irAEs during cancer immunotherapy.

## 1 Introduction

Rheumatological toxicity during immunotherapy has a highly variable incidence depending on the case considered, estimated to be around 10% of treated patients (1). Although not precise, published studies have documented different incidence rates of Sjögren’s syndrome in patients treated with immune checkpoint inhibitors (ICIs), ranging from 1.2% to 24.2% (2). Le Burel et al. showed that in a sample of 908 patients, the estimated prevalence of Sjögren’s syndrome as an immune-related adverse event in patients treated with anti-PD1/anti-PD-L1 agents alone or in combination with anti-PD1 and anti-CTLA-4 agents was 0.3% and 2.5%, respectively (3).

The main clinical presentations described include unspecified arthralgia and myalgia (including mechanical arthralgia), inflammatory arthropathies/arthritis, polymyalgia-like syndromes, sicca syndrome and myositis; more rarely, cases of eosinophilic fasciitis, vasculitis and sporadic cases of systemic sclerosis, digital ischaemia, sarcoidosis and lupus nephritis have been reported (4). The syndromes described only partially reflect classic rheumatic diseases and differ from them in some features, including a different gender distribution and lower autoantibody seropositivity (5).

**Figure 1:**
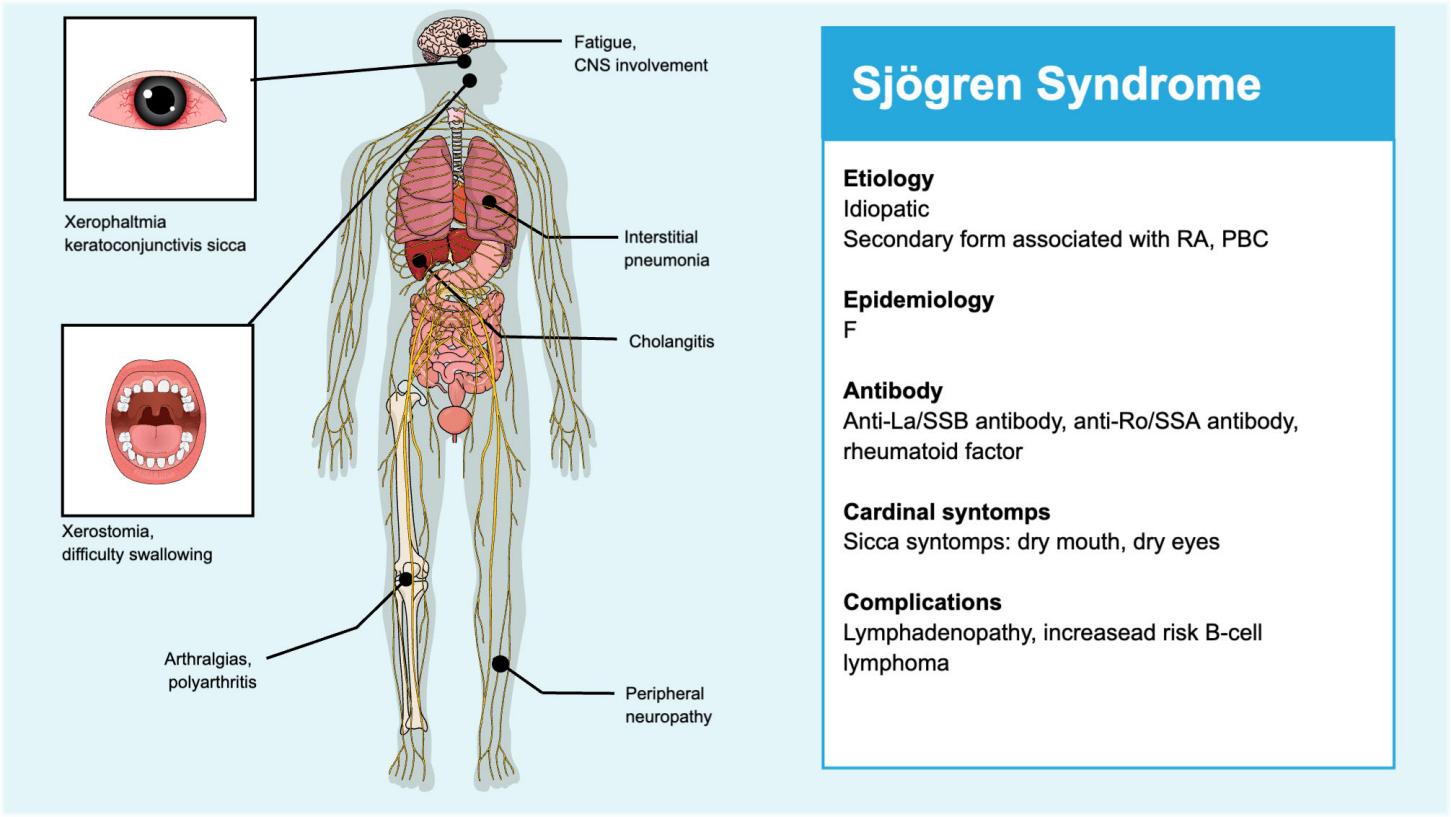
Symptoms of Sjögren's syndrome.

Sjögren’s syndrome is a common chronic systemic inflammatory disease of autoimmune nature and unknown aetiology. Its clinical presentation is characterised by dryness of the mouth, eyes and other mucous membranes resulting from lymphocytic infiltration of the exocrine glands and subsequent secondary dysfunction. This syndrome has the potential to affect various exocrine glands or other organs (6) (**Figure 1**).

The cause of Sjögren’s syndrome (SjS) due to immunotherapy is not fully understood, but blocking the PD-1/PD-L1 pathway with immune checkpoint inhibitors (ICIs) appears to trigger T-cell activation, leading to infiltration of the salivary gland epithelium (7). Pringle et al. proposed that ICIs induce inflammation via IFN-γ, suggesting a new type of type II interferonopathy (8).

Here we report a clinical case of atezolizumab-induced Sjögren’s syndrome (SjS) in triple-negative breast cancer (TNBC). To our knowledge, this is the first clinical case reported in the literature in which we successfully achieved early diagnosis of SjS during treatment with immune checkpoint inhibitors (ICIs) without treatment interruption, resulting in almost complete clinical resolution of the adverse event.

## 2 Case description

**Figure 2:**
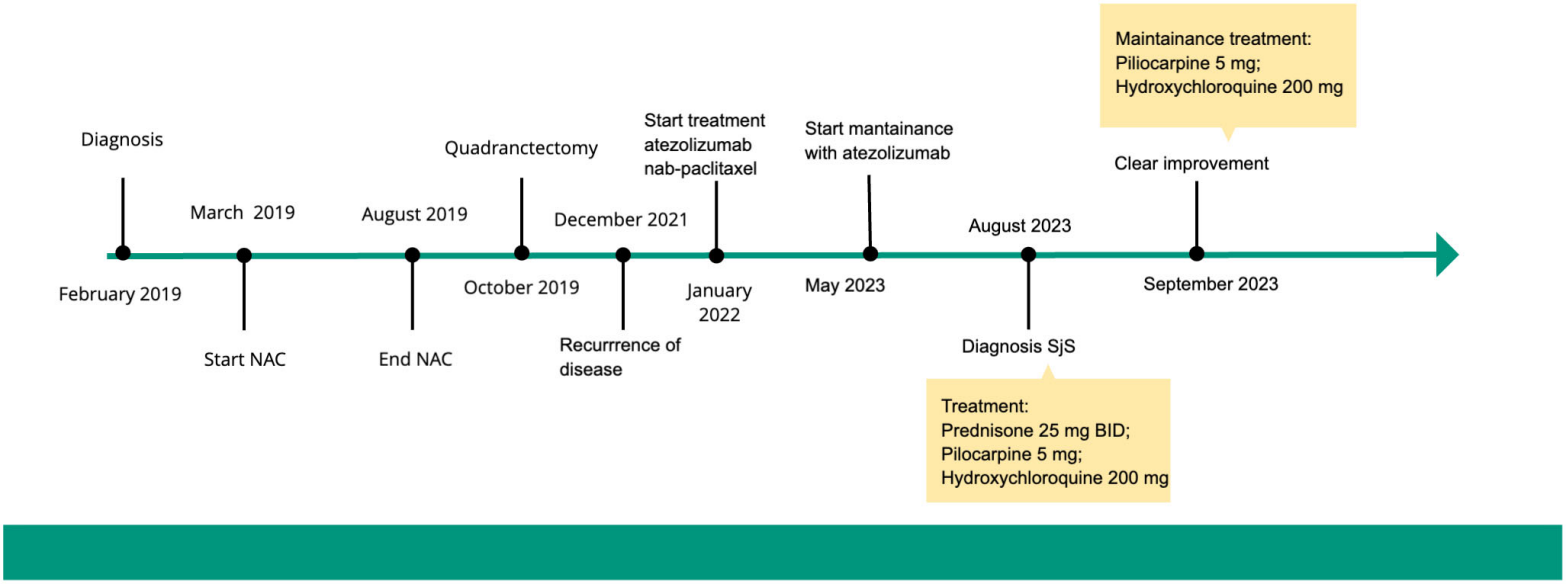
Timeline.

A 40-year-old female patient was diagnosed with triple-negative ductal carcinoma of the left breast in February 2019. She was treated with neoadjuvant chemotherapy (NAC), followed by quadrantectomy and sentinel node biopsy in October 2019, with a pathological staging of ypT1c ypN0 pNR (**Figure 2**).

Subsequently, she underwent radiation therapy (RT) to the left breast, with a total dose of 42.4 Gy in 16 fractions, plus a scar boost for a total dose of 10 Gy (January 2020).

The patient continued to have regular clinical and instrumental follow-up until a CT scan was performed in September 2022, which revealed the appearance of a solid, non-calcified nodule measuring 13 mm in the medial segment of the LM. A PET/CT FDG scan demonstrated the well-characterised radiological solid lung nodule with high FDG uptake (SUV max 31) and intense radioconcentration (SUV max 41) in the pre/subcarinal lymph node area.

A CT-guided lung biopsy was performed in December 2021, which revealed a site of carcinoma infiltration within the lung parenchyma, characterised by solid nests that were compatible with a primary mammary tumour (GATA3+, TTF1-). Immunohistochemistry (IHC) revealed the absence of estrogen (ER) and progesterone (PGR) receptor expression in 100% of tumour nuclei. The HER2 score was 0, and the tumour proliferative index, as determined by MIB-1 expression, was 70%.

We decided to start treatment with atezolizumab at a dose of 840 mg administered intravenously on days 1 and 15 and nab-paclitaxel at a dose of 100 mg per square metre of body surface area on days 1, 8 and 15 of each 28-day cycle (9). After thirteen cycles of atezolizumab and nab-paclitaxel, the patient remained in partial remission. The regimen was well tolerated by the patient. The most common adverse events reported were fatigue and peripheral neuropathy. To improve her quality of life and reduce the number of infusion centre visits, we decided to discontinue nab-paclitaxel and continue atezolizumab at 1200 mg every three weeks as maintenance therapy (May 2023).

Following four cycles, the patient initially presented with a dry, burning sensation in her throat. Subsequently, the oropharyngeal symptoms became progressively worse, resulting in an increasing difficulty in swallowing due to severe dryness of the mucous membranes and a lack of salivation, as reported by the patient. On examination, the patient’s tongue was observed to have a dark brown appearance, and there was a notable degree of dryness of the buccal mucosa. An empirical treatment regimen comprising mouthwash and fluconazole 50 mg daily was initiated, based on the clinical suspicion of an oral fungal infection. However, these therapeutic measures proved ineffective in alleviating the symptoms. Additionally, the patient reported a progressive deterioration in her dry eye condition, an alteration in her taste perception, and the onset of arthralgia. An ophthalmological examination revealed significant xerophthalmia, as indicated by the Schirmer test, and the presence of corneal erosions was also documented.

The definitive diagnosis of SjS was established based on the presence of three positive results in accordance with the 2016 ACR-EULAR Classification Criteria for Sjögren’s Syndrome (10). These were: 1) decreased salivary secretion by the Saxon test (0.3 g/2 min); 2) decreased Tear secretion was also assessed via Schirmer’s test, with the right eye recording 8 mm/5 min and the left eye 2 mm/5 min. Additionally, the anti-RO 2 level was found to be >1374.8, while the anti-RO60 level was 1275.4, the anti-La level was >1550 A, and the rheumatoid factor level was 61.

A two-week oral treatment regimen comprising prednisone 25 mg twice a day, in conjunction with pilocarpine 5 mg and hydroxychloroquine 200 mg, was initiated without interruption to the immunotherapy (August 2023).

Following a one-month course of treatment, the patient reported a notable improvement in previously documented symptoms. Consequently, the steroid therapy was adjusted, and the treatment regimen was maintained with hydroxychloroquine and pilocarpine.

To date, the patient continues therapy with Atezolizumab with substantial SD and good QoL.

## 3 Discussion

To the best of our knowledge, no individual cases of ICI-induced Sjögren’s syndrome in TNBC have been previously described in the literature. The diagnosis of SjS as an irAE was confirmed three months after the onset of symptoms, despite the administration of prednisolone and pilocarpine hydrochloride, which resulted in symptomatic improvement.

ICI-associated sicca syndrome (ICI-sicca) has been extensively documented, with an incidence range of 3% to 24% across clinical trials, and no discernible correlation with specific tumour types or ICI agents (11). Clinically, ICI-sicca has been reported to have an abrupt onset and may have a greater prevalence of oral versus ocular involvement. Furthermore, the sicca symptoms often respond to corticosteroids, in contrast to classic SjS (12).

Laboratory features of SjS may include cytopenias, hypocomplementemia, cryoglobulinemia, monoclonal gammopathy, elevated ESR, and autoantibodies including ANA, anti-Ro/SSA, anti-La/SSB, and RF (13).

The initial documentation of Sjögren’s syndrome induced by ICI was observed in four cases (0.5%) among a cohort of 700 patients. Cappelli et al. reported on 13 patients referred by oncologists to the Johns Hopkins Rheumatology clinics from 2012 to 2016 because they had been identified as having new rheumatological symptoms in the context of treatment with ipilimumab and/or nivolumab for NSCLC, MM or renal cell carcinoma. Four patients exhibited sicca symptoms, with all four presenting with a relatively abrupt onset of severe dry mouth and severe salivary hypofunction. The patient also exhibited concurrent bilateral parotid gland enlargement, which was observed to regress with the administration of steroid therapy. Three of the patients presenting with sicca symptoms had antinuclear antibodies (ANA), one patient had anti-La (SSB) antibodies, and one patient was rheumatoid factor (RF)-positive (14).

In a systematic literature review, the same author (Cappelli) identified 52 papers containing information about musculoskeletal or rheumatic irAEs as a consequence of ICPI treatment. Of the 52 papers identified, 33 were clinical trials, 3 were observational studies, and 16 were case reports or series. A single patient treated with nivolumab was observed to present with concomitant sicca syndrome and tubulointerstitial nephritis (15).

Le Burel et al. conducted a screening of the French prospective multicentre academic oncologic registry, the REISAMIC registry, with the objective of recruiting cases of grade ≥ 2 irAEs occurring in ICPI-treated patients. This was done in order to study the prevalence of irAEs in patients treated with anti-PD1/PDL1. A total of 21 patients, out of 908 individuals treated with anti-PD1/PDL1 (in conjunction with an anti-CTLA-4 agent in 40 cases), experienced systemic rheumatologic irAEs, including SjS (0.3%). A greater proportion of patients with SjS had received combination therapy with ipilimumab (2.5%) (3).

Ramos Casals et al. identified 26 patients who had undergone treatment with PD-1/PD-L1 inhibitors. The primary SjS-related characteristics observed were xerostomia in 25 (96%) patients, dry eye in 17 (65%), abnormal ocular tests in 10/16 (62%) and abnormal oral diagnostic tests in 12/14 (86%) patients. A minor salivary gland biopsy was performed in 15 patients. The histopathological findings consisted of mild chronic sialadenitis in eight patients (53%) and focal lymphocytic sialadenitis in the remaining seven patients (47%). A focus score was measured in five of the six patients (mean of 1.8, range 1-4). Immunological markers were present in 13 of the 25 patients (52%), including positive anti-nuclear antibodies (ANA) in 13 patients, anti-Ro/SS-A in 5 patients, rheumatoid factor (RF) in 2 patients, anti-La/SS-B in 2 patients, low complement 3 (C3) and 4 (C4) levels in 1 patient each, and positive cryoglobulins in 1 patient. The multicentric retrospective study revealed a male gender predominance, increased extraglandular involvement, and a decreased frequency of autoantibodies in comparison to classic Sjögren’s syndrome (5).

Warner BM et al. observed a predominant T‐lymphocytic infiltrate in salivary gland biopsy specimens, with a slight majority of CD4+ over CD8+ T cells and few CD20+ B cells. This pattern differs from that observed in typical SS infiltrates, where B cells comprise 20%–62% of all lymphocytes and FS is directly correlated with B-cell ratios. The targets of ICI therapies (e.g., PD-1 and PD-L1) demonstrated variable positivity in patients undergoing ICI. Scattered PD-1-positive T cells and epithelial PD-L1 positivity were observed, though these were limited to the most severely infiltrated cases (11).

Calabrese et al. presented a series of patients evaluated at the Cleveland Clinic Foundation from 2015 to 2016 with rheumatic irAEs resulting from cancer immunotherapy, as well as patients with a pre-existing autoimmune rheumatic diseases (ARDs) who were evaluated pre-emptively. In the cohort of 13 patients without a prior diagnosis of ARD, five individuals developed sicca syndrome. The median time to onset of irAE was 7.3 weeks (range 2–48.4). Autoimmune testing revealed the presence of various autoantibodies, including anti-nuclear antibodies (ANA), anti-double-stranded DNA (anti-dsDNA), rheumatoid factor (RF), and anti-Sjögren’s syndrome antigen A (anti-SSA (Ro)) in select cases (16).

Other authors provide a more detailed account of rheumatic irAEs, describing a broader range of rheumatic irAEs than previously observed. These include inflammatory arthritis, sicca syndrome, a PMR-like syndrome and myositis in patients undergoing monotherapy and combination therapy with checkpoint inhibitors (16, 17).

The precise mechanism by which SjS is caused by immunotherapy remains unclear. However, the impairment of the PD-1/PD-L1 pathway by ICIs appears to result in the activation of T-lymphocytes, which in turn leads to the infiltration of salivary gland epithelium (18). Pringle et al. proposed that ICIs are induced by IFN-γ and represent a novel form of type II interferonopathy (8).

A multidisciplinary team comprising at least one oncologist and one rheumatologist is essential for the informed decision-making process in patient care. The input of a rheumatologist is of particular value in the context of autoimmune diseases, where their expertise in understanding the symptomatology of such conditions and in managing rheumatic immune-related adverse events (irAEs) is of great benefit. This is especially the case in instances where immunosuppressive agents and biologics are required for patients with steroid-refractory outcomes. Furthermore, the patient’s preferences must be taken into account in formulating the final treatment plan (1). Once the diagnosis has been confirmed, the severity of the disease should be classified in accordance with the Common Terminology Criteria for Adverse Events (CTCAE version 5.0) (19), a standard framework for oncologists. However, this classification system is not typically employed by rheumatologists. It is therefore inappropriate to base a referral to a rheumatologist on the classification alone (20).

It is recommended that any suspicion of rheumatic irAEs, in the absence of improvement following initial treatment with nonsteroidal anti-inflammatory drugs (NSAIDs), should prompt referral to a rheumatologist, even in cases of mild adverse events, such as grade 1 (G1). This approach facilitates timely diagnosis and the initiation of treatment, which differs from the management of other irAEs (20). Rheumatologists have established their own criteria for characterising adverse events in clinical trials, taking into account factors such as symptom duration, lifestyle modifications, and treatment effects (21). Nevertheless, these criteria are not widely acknowledged or employed by oncologists. As with other irAEs, it is of the utmost importance to achieve a balance between the effective treatment of tumours through immunotherapy and the management of irAEs through immunosuppression (20).

Corticosteroid treatment was observed to result in symptom improvement in the majority of cases of sicca syndrome induced by various ICIs (22). Neither interruption nor discontinuation of ICIs was necessitated by the occurrence of oral symptoms. Although SjS is not typically a life-threatening condition, it has the potential to significantly impact the quality of life of those affected. In cases where patients develop SjS while undergoing ICIs, a case-by-case multidisciplinary approach is recommended, as ICIs may potentially impact tumour response and/or overall survival (23).

In order to assess the impact of high-dose oral corticosteroids (0.5–1 mg/kg/day) on the management of this irAE, it is necessary to obtain real-world data with objective measures, with a view to defining the optimal dose and duration of treatment (24).

In this case, atezolizumab demonstrated therapeutic efficacy and maintained tumour shrinkage, indicating a potential correlation between irAE development and therapeutic outcome (25).

## 4 Conclusion

This report details a case of clinical Sjögren’s syndrome (SjS) induced by atezolizumab in a patient with triple-negative breast cancer (TNBC). Given the significant impact of SjS on patients’ quality of life, it is imperative that oncologists exercise diligence in the recognition of any indications of salivary gland hypofunction and engage in collaboration with specialists from a range of disciplines, including ophthalmology, otolaryngology, and rheumatology.

A multidisciplinary and interdisciplinary approach is of paramount importance for the expeditious diagnosis and treatment of rheumatic immune-related adverse events (irAEs). Further research is required in order to establish the optimal management strategies for these conditions.

## Data Availability

The raw data supporting the conclusions of this article will be made available by the authors, without undue reservation.

## References

[1] Kostine M Finckh A CO B Visser K Leipe J Schulze-Koops H. EULAR points to consider for the diagnosis and management of rheumatic immune-related adverse events due to cancer immunotherapy with checkpoint inhibitors. Ann Rheum Dis. (2021) 80:36–48. doi: 10.1136/annrheumdis-2020-217139 32327425 PMC7788064

[2] Abdel-Rahman O Oweira H Petrausch U Helbling D Schmidt J Mannhart M. Immune-related ocular toxicities in solid tumor patients treated with immune checkpoint inhibitors: a systematic review. Expert Rev Anticancer Ther. (2017) 17:387–94. doi: 10.1080/14737140.2017.1296765 28277102

[3] Le Burel S Champiat S Mateus C Marabelle A JM M Robert C. Prevalence of immune-related systemic adverse events in patients treated with anti-Programmed cell Death 1/anti-Programmed cell Death-Ligand 1 agents: a single-centre pharmacovigilance database analysis. Eur J Cancer. (2017) 82:34–44. doi: 10.1016/j.ejca.2017.05.032 28646772

[4] Dang QM Watanabe R Shiomi M Fukumoto K TW N Okano T. Rheumatic immune-related adverse events due to immune checkpoint inhibitors-A 2023 update. Int J Mol Sci. (2023) 15;24:5643. doi: 10.3390/ijms24065643 PMC10051463 36982715

[5] Ramos-Casals M Maria A ME Suárez-Almazor Lambotte O BA F Hernàndez-Molina G. Sicca/Sjögren’s syndrome triggered by PD-1/PD-L1 checkpoint inhibitors. Data from the International ImmunoCancer Registry (ICIR). Clin Exp Rheumatol.;. (2019) 37 Suppl 118:114–22. 31464670

[6] Ramos-Casals M Brito-Zerón P Bombardieri S Bootsma H De Vita S Dörner T. EULAR-Sjögren Syndrome Task Force Group. EULAR recommendations for the management of Sjögren’s syndrome with topical and systemic therapies. Ann Rheum Dis. (2020) 79:3–18. doi: 10.1136/annrheumdis-2019-216114 31672775

[7] Shen P Deng X Hu Z Chen Z Huang Y Wang K. Rheumatic manifestations and diseases from immune checkpoint inhibitors in cancer immunotherapy. Front Med. (2021) 8:762247. doi: 10.3389/fmed.2021.762247 PMC8599930 34805229

[8] Pringle S Wang X Vissink A Bootsma H Kroese FGM. Checkpoint inhibition-induced sicca: A type II interferonopathy? Clin Exp Rheumatol. (2020) 38 Suppl 126:253–60. 33025881

[9] Schmid P Adams S HS R Schneeweiss A CH B Iwata H. IMpassion130 trial investigators. Atezolizumab and nab-paclitaxel in advanced triple-negative breast cancer. N Engl J Med. (2018) 29;379:2108–21. doi: 10.1056/NEJMoa1809615 30345906

[10] Shiboski CH SC S Seror R LA C Labetoulle M TM L. 2016 american college of rheumatology/european league against rheumatism classification criteria for primary sjögren’s syndrome: A consensus and data-driven methodology involving three international patient cohorts. A. rthritis Rheumatol. (2017) 69:35–45. doi: 10.1002/art.39859 PMC5650478 27785888

[11] Warner BM AN B EJ L Allen C Hinrichs C Rajan A. Sicca syndrome associated with immune checkpoint inhibitor therapy. Oncologist. (2019) 24:1259–69. doi: 10.1634/theoncologist.2018-0823 PMC6738284 30996010

[12] Conde-Flores E Remolina-Bonilla Y Castro-Alonso F Martínez-Ibarra N Hernández-Molina G Chapa-Ibargüengoitia M. Sjögren syndrome induced by immune checkpoint inhibitors in a patient with advanced renal cell carcinoma. Oncol (Williston Park). (2021) 14;35:486–90. doi: 10.46883/ONC.2021.3508.0486 34398593

[13] Ladouceur A Ezdoglian A JA S Hudson M Jamal S Clifford A. The utility of laboratory investigations for the assessment and management of rheumatic immune related adverse events. Rheum Dis Clin North Am. (2024) 50:181–99. doi: 10.1016/j.rdc.2024.01.003 38670720

[14] Cappelli LC AK G AN B Albayda J RL M Haque U. Inflammatory arthritis and sicca syndrome induced by nivolumab and ipilimumab. Ann Rheum Dis. (2017) 76:43–50. doi: 10.1136/annrheumdis-2016-209595 27307501 PMC5333990

[15] Cappelli LC AK G Bingham CO 3rd Shah AA. Rheumatic and musculoskeletal immune-related adverse events due to immune checkpoint inhibitors: A systematic review of the literature. Arthritis Care Res (Hoboken). (2017) 69:1751–63. doi: 10.1002/acr.23177 PMC5478477 27998041

[16] Calabrese C Kirchner E Kontzias A Velcheti V Calabrese LH. Rheumatic immune-related adverse events of checkpoint therapy for cancer: Case series of a new nosological entity. RMD Open.;. (2017) 3:e000412. doi: 10.1136/rmdopen-2016-000412 28405474 PMC5372131

[17] Mavragani CP Moutsopoulos HM. Sicca syndrome following immune checkpoint inhibition. Clin Immunol. (2020) 217:108497. doi: 10.1016/j.clim.2020.108497 32531346

[18] Christodoulou MI EK K Moutsopoulos HM. Characteristics of the minor salivary gland infiltrates in Sjögren’s syndrome. J Autoimmun. (2010) 34:400–7. doi: 10.1016/j.jaut.2009.10.004 19889514

[19] Common Terminology Criteria for Adverse Events (CTCAE) v5.0 . Available online at: https://ctep.cancer.gov/protocoldevelopment/electronic_applications/docs/CTCAE_v5_Quick_Reference_5x7.pdf (Accessed 21 July 2022).

[20] Pacholczak-Madej R Kosałka-Węgiel J Kuszmiersz P JW Mituś Püsküllüoğlu M Grela-Wojewoda A. Immune checkpoint inhibitor related rheumatological complications: cooperation between rheumatologists and oncologists. Int J Environ Res Public Health. (2023) 10;20:4926. doi: 10.3390/ijerph20064926 PMC10049070 36981837

[21] Woodworth T DE F Alten R Bingham CO 3rd Yocum D Sloan V. Standardizing assessment and reporting of adverse effects in rheumatology clinical trials II: the Rheumatology Common Toxicity Criteria v.2.0. J Rheumatol. (2007) 34:1401–14. 17552067

[22] Brahmer JR Lacchetti C BJ S MB A KJ B JM C. Management of immune-related adverse events in patients treated with immune checkpoint inhibitor therapy: American society of clinical oncology clinical practice guideline. J Clin Oncol. (2018) 36:1714–68. doi: 10.1200/JCO.2017.77.6385 PMC6481621 29442540

[23] Haanen J Obeid M Spain L Carbonnel F Wang Y Robert C. ESMO Guidelines Committee. Management of toxicities from immunotherapy: ESMO Clinical Practice Guideline for diagnosis, treatment and follow-up. Ann Oncol. (2022) 33:1217–38. doi: 10.1016/j.annonc.2022.10.001 36270461

[24] Reid P Cappelli LC. Treatment of rheumatic adverse events of cancer immunotherapy. Best Pract Res Clin Rheumatol. (2022) 36:101805. doi: 10.1016/j.berh.2022.101805 36539321 PMC10198805

[25] Kostine M Rouxel L Barnetche T Veillon R Martin F Dutriaux C. Rheumatic disorders associated with immune checkpoint inhibitors in patients with cancer-clinical aspects and relationship with tumour response: a single-centre prospective cohort study. Ann Rheum Dis. (2018) 77:393–8. doi: 10.1136/annrheumdis-2017-212257 29146737

